# Effects of the passive self-ligating system on alveolar bone thickness, dental inclinations, and arch dimensions

**DOI:** 10.4317/jced.61845

**Published:** 2024-11-01

**Authors:** Murilo Matias, Bruno Vieira, Karina Maria Salvatore Freitas, Célia Regina Maio Pinzan-Vercelino, Paula Cotrin, Fabrício Pinelli Valarelli, Diana Margarita Pirovano Caceres, Guilherme Janson, Marcos Roberto de Freitas

**Affiliations:** 1 D.D.S., M.Sc., Ph.D. Associate Professor. Department of Orthodontics. Guarulhos University, UNG, São Paulo, Brazil; 2 D.D.S., M.Sc., Ph.D. Private Practice. Belo Horizonte, Minas Gerais, Brazil; 3 D.D.S., M.Sc., Ph.D. Associate Professor. Department of Orthodontics, Ingá University Center UNINGÁ, Maringá, PR, Brazil; 4 D.D.S., M.Sc., Ph.D. Associate Professor. Department of Orthodontics. Ingá Dental School, Maringá, Paraná, Brazil; 5 M.Sc Student. Department of Orthodontics, Ingá University Center UNINGÁ, Maringá, PR, Brazil; 6 D.D.S., M.Sc., Ph.D. Professor. Department of Orthodontics. Bauru Dental School, University of São Paulo, Brazil

## Abstract

**Background:**

We aimed to evaluate changes in buccal bone thickness (BBT), buccolingual dental inclinations (BLI), and transversal widths (TW) after treatment using a passive self-ligating system.

**Material and Methods:**

Pre- and posttreatment cone-beam computed tomography images (CBCT) of 21 Class I patients (initial mean age: 14.99 ± 1.27 years; initial crowding of at least 4mm) treated without extractions using passive self-ligating appliances were evaluated. Buccal bone thickness, dental inclinations, and transversal widths were measured, and their changes were compared using paired t-test. The associations were assessed using the Pearson correlation coefficient.

**Results:**

BBT showed statistically significant decreases in both arches, mainly for the posterior teeth. Most teeth were proclinated after treatment, with more buccal tipping occurring for the anterior teeth and second premolars in both arches. The results demonstrated significant increases in maxillary and mandibular TW, except for maxillary intercanine distance. Negative correlations between BBT and buccal inclination were observed for the maxillary right lateral incisor, maxillary left second premolar, right mandibular canine, and between BBT and TW for the maxillary left second premolar. A positive association was observed between BBT and TW only for the mandibular right first premolar.

**Conclusions:**

In general, the treatment with passive self-ligating system showed an expansion of the dental arches followed by a decrease in BBT, probably caused by buccal inclination in both arches.

** Key words:**Orthodontic Brackets, Cone-Beam Computed Tomography, Periodontium, Orthodontics.

## Introduction

Orthodontic objectives include pleasant facial esthetics, an efficient masticatory system, sTable treatment results, and healthy dental and periodontal tissues. The influences of orthodontic treatment on the gingiva, marginal periodontium, attachment levels, and root integrity have been previously discussed (1,2). In adult orthodontic treatment, the focus is on the aging processes of the periodontal ligament and a varying degree of alveolar bone loss. This periodontal involution increases the risk of bone dehiscence, fenestrations, and root resorption. Animal experiments have shown that orthodontic tooth movement may induce bone plate loss (1).

The philosophy of the self-ligating appliance is based on using low forces to obtain tooth movement. The pressure must be low enough to prevent blood vessels from occluding, allowing the cells and the necessary biochemical messengers to be transported to the areas where bone remodeling will occur, allowing the tooth to be moved. Beyond those advantages, self-ligating appliances have stated the possibility of increasing the dental arch dimensions without periodontal damage, claiming that alveolar bone would follow the tooth movement. It is suggested that a new bone could be reshaped at the side which the tooth is being moved because more biocompatible forces are used in this system due to the combination of self-ligating brackets with high-tech and high-resilient copper–nickel–titanium archwires because of the light forces delivered and low friction. According to Damon (3), the arch dimension increase is achieved by dental bodily movement with minimal tipping, with alveolar bone and surrounding tissues remodeling.

Class I nonextraction treatment of crowded teeth without stripping, extraction, or skeletal expansion requires arch perimeter increase for crowding alleviation, so transverse dental expansion and proclination may occur. Some studies have suggested that dental proclination raises the risk of alveolar bone defects (4) and gingival recession (5). Thus, a nonextraction protocol that proposes an increase in the dimensions of dental arches should be carefully evaluated.

Cone-bean computed tomography (CBCT) development facilitates the evaluation of human bone structures since it shows accuracy for dental and bone measurements (6). Previous CBCT studies evaluated the effects of the self-ligating system only after the leveling and alignment phase (7,8). Recently, a study (9) using CBCT images demonstrated marginal bone loss in alveolar buccal bone height. Few studies (10,11) evaluated the entire treatment using CBCT, but not all the teeth were investigated. Moyano *et al*. (12) evaluated models and lateral cephalograms, and Lucchese *et al*. (13) evaluated dental movements and stability of the corrections using 3-dimensional (3D) analysis. However, there is still a lack of evidence regarding using CBCT to evaluate bone and dental changes in both arches after complete orthodontic treatment using a self-ligating system. Therefore, the present study aimed to assess, by CBCT, changes in the buccal bone thickness, buccolingual dental inclinations, and transversal widths in the maxillary and mandibular arches after completed nonextraction treatment using a passive self-ligating system.

## Material and Methods

The local institutional review board approved this study (protocol number: 1.567.401). Consent to undergo the CBCT exams and to use the material for the present investigation was obtained from all patients or parents/legal guardians.

The sample size was calculated based on an alpha significance level of 0.05 and a beta of 0.2 to achieve 80% power to detect a mean difference of 1.06mm, with a standard deviation of 1.64 for the buccal bone thickness (7). The sample size calculation showed that 20 patients were needed. According to this result, 21 patients were selected.

The following inclusion criteria were adopted: Class I patients treated with a passive self-ligating system; from 11 to 17 years old; with no previous history of orthodontic treatment; anterior crowding of at least 4mm; absence of crossbite; complete permanent dentition up to second molars; without agenesis or dental losses, impacted or supernumerary teeth.

The exclusion criteria included patients with posterior crossbite and CBCT images suggesting periodontal diseases such as horizontal or vertical proximal bone loss, furcal involvement, and calculus. Teeth with extensive restorations involving the cementoenamel junction were also excluded from the evaluation.

All patients were treated with the 0.022-in Damon 3MX passive self-ligating appliance, standard prescription, according to the guidelines of the Damon system (Ormco Corporation, Orange, CA, USA). The archwire sequence included an initial 0.014-in Damon Copper NiTi (left in place an average of 12 weeks) followed by a 0.014 × 0.025-in Damon Copper NiTi (12–18 weeks). The finishing archwire used was 0.019 × 0.025-in SS coordinated according to the arch form obtained after inserting the 0.014 x 0.025-in CuNiTi Damon archwire. No other appliances were used, such as anchoring devices, expanders, functional appliances, lip bumper, or distalizing appliances. The appliances were removed when a Class I canine-molar relationship and ideal overjet and overbite were achieved, and posttreatment records were taken.

CBCT images were taken at the pre- (T1) and posttreatment (T2) stages using i-CAT Classic scanner (Imaging Sciences International, Hatfield, Pa). The subjects were positioned with the Frankfurt horizontal plane parallel to the floor and instructed to maintain maximum intercuspation with the tongue touching the palate and to avoid swallowing during the scanning period. The imaging protocol used was 120 kV, 5 mA, 13 x 16 cm field of view (FOV), 0.25 mm voxel size, and a scanning time of 40 seconds. Five CBCT final images were taken with a 3D Accuitomo-XYZ Slice View Tomography (J. Morita, Kyoto, Japan) with 90 kV, 7 mA, 13 x 16 cm FOV, 0.20 mm voxel size and a scanning time of 30 seconds, with the patient following the same positioning protocol.

Images were saved in DICOM files and imported into Dolphin 3-dimensional software (version 11.9; Dolphin Imaging and Management Solutions, Chatsworth, Calif) to obtain the primary reconstructed images (sagittal, coronal, and axial) and 3D reconstructions.

**Figure 1:**
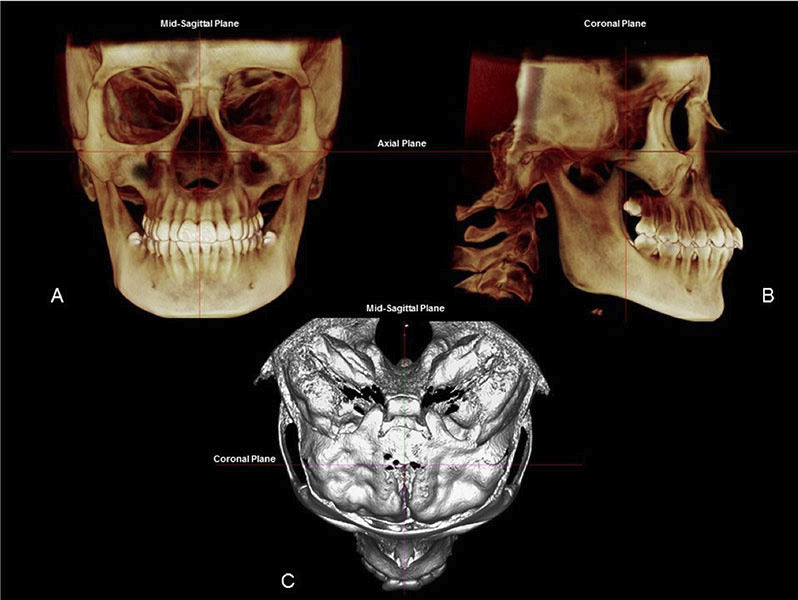
Head position standardization: frontal view (A), sagittal view (B), and axial view (C).

Before image selection for measurement, the head was standardized in all three planes. The standardization of head position was performed in 3D images: in frontal view, the infraorbital plane (plane formed by the points located in the upper region of infraorbital foramen) parallel to the floor; in sagittal view, the Frankfurt horizontal plane (the plane defined bilaterally by the right and left porion and right and left orbitale landmarks) parallel to the floor; and in axial view, the midsagittal line (center point in foramen magnum to point located on the crista galli) was perpendicular to the floor. Head orientation was the same for each CBCT image performed by the same operator (Fig. 1).

All measurements were performed using the tools provided by Dolphin Imaging Software. Only one calibrated examiner (MM) evaluated all sectional images in a dark room using a 224.1-inch LCD monitor with a 1920 x 1200 pixels resolution.

-Buccal bone thickness (BBT) measurements:

Measurements of BBT were performed by selecting the coronal and axial visualization displays.

**Figure 2:**
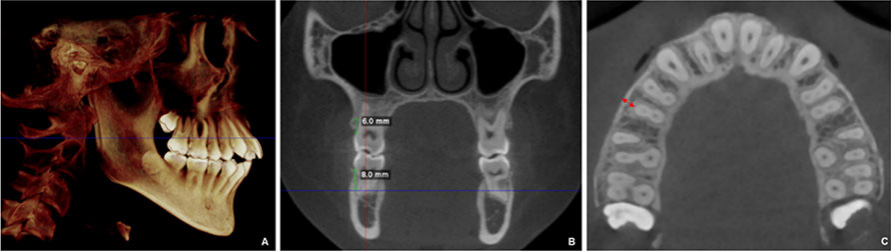
Buccal bone thickness measurements. Selection of the height of the measurement in the maxilla (blue horizontal line) in sagittal view (A); measurements performed 6mm (in the maxilla) and 8 (in the mandible) from the cementoenamel junction in the apical direction in the coronal view (B); measurements of buccal bone thickness performed from buccal limit of root contour to the buccal surface of the cortical plate, perpendicularly to the dental arch (C).

The next step involved magnifying the sagittal view and selecting the level of the measurement (in the maxilla or mandible), as indicated by the blue horizontal lines. The measures were performed at 4 and 6mm (in the maxilla) and 4 and 8mm (in the mandible) from the cementoenamel junction of the right maxillary/mandibular molar in the direction of the apical area, as seen in the coronal view. The measurements of BBT were performed from the buccal limit of root contour to the buccal surface of the cortical plate, perpendicularly to the dental arch, for all teeth. The images were amplified for visualization (Fig. 2).

**Figure 3:**
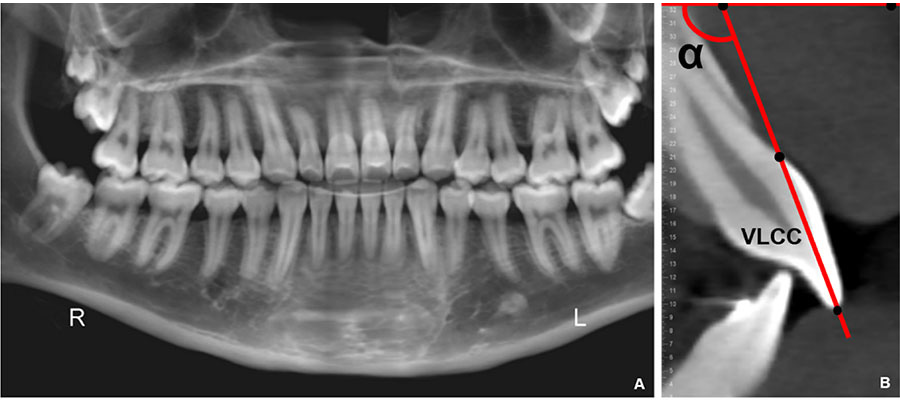
Buccolingual inclination measurements. CBCT-panoramic reconstruction (A); angle formed by the buccal line of the clinical crown (BLCC) until the upper edge of the selected image (B).

-Buccolingual inclination (BLI) measurements:

The panoramic reconstruction was used for the measurements of the buccolingual inclinations (BLIs). In the panoramic reconstruction, cross-sections were made to determine the best view of the buccal face of the clinical crown of each tooth, from the angle formed by the buccal line of the clinical crown (BLCC: line formed by the points located in the most incisal/occlusal region of the buccal surface of the clinical crown and cementoenamel junction; the point used for delimitation of the clinical crown in posterior teeth was the tip of the buccal cusp.) and the upper or lower edge of the selected image, in the panoramic cross-section (Fig. 3).

**Figure 4:**
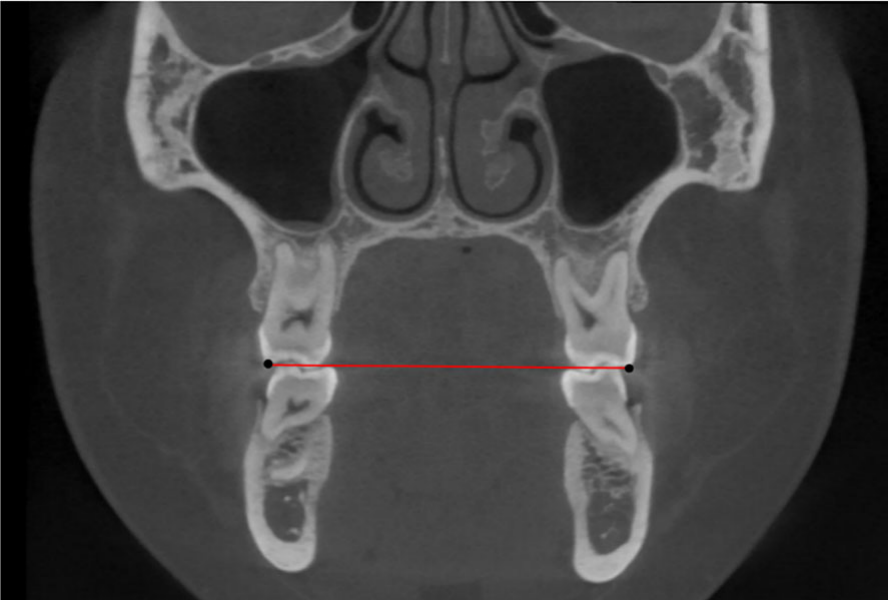
Transverse measurement in the coronal plane of the 2D image.

-Transversal width (TW) measurements:

For intermolar, inter-premolar, and intercanine distance measurements, coronal images from CBCT scans were used. The images selected from the maxillary and mandibular arches were obtained from the teeth’ complete visualization. To measure the transverse distances, buccal cusp tips were selected for maxillary and mandibular canine, first, and second premolars, while mesiobuccal cusp tips were chosen for the first and second molars (Fig. 4). Crowding was measured using Little’s Irregularity Index (14) in the initial digital casts for maxillary and mandibular arches.

-Error study:

To evaluate the error of the method, CBCT scans of 20% of the sample, randomly selected, were re-evaluated after 30 days of the first measurement to verify the reproducibility of the method. The systematic error was calculated using the dependent t-test and the casual error according to Dahlberg’s formula (S2 = Σd2/2n) for *p* < 0.05.

-Statistical Analyses:

Data were tested for normal distribution using the Shapiro-Wilk test. As data were normally distributed, parametric tests were applied.

The differences between post- (T2) and pretreatment (T1) mean values were calculated, and the treatment changes of BBT, BLI, and TW were evaluated using paired t-tests. The associations among BBT, BLI, and TW were analyzed using the Pearson correlation coefficient.

All statistical analyses were performed using SPSS software (version 20; IBM Corporation, Armonk, USA). Results were considered statistically significant at *p*<0.05.

## Results

No systematic errors were detected (BBT: *p*= 0.54; BLI: *p*= 0.69; TW: *p*= 0.06), casual errors varied from 0.16 mm to 0.35 mm to BBT and TW, respectively, and it was 0.880 for BLI, and are within acceptable levels. 

Patients’ demographic distribution was described in the following aspects: initial and final mean age, treatment time, and Little irregularity index ( Table 1).

For the maxillary arch at 4 mm level, there were statistically significant decreases in BBT for right and left first molars and second premolars, right lateral incisor, and left first premolar. In the mandibular arch, there were statistically significant decreases in BBT for the left first molar and right second molar ( Table 2).

There were statistically significant decreases in BBT for the maxillary arch at 6 mm level for the right and left first molars. In the mandibular arch, statistically significant decreases in BBT at 8 mm level were observed for the left second premolar and right first molar ( Table 3).

There were statistically significant increases in buccal inclination of all incisors, right and left first and second premolars in the maxillary arch. The upper right canine and upper and left first and second molars showed a slight decrease in buccal inclination. No change in buccal inclination was observed for the left canine. In the mandibular arch, statistically significant increases in buccal inclination were observed for most teeth, except for the left first molar, whose change was slight, and the left canine, which almost remained with de pretreatment inclination ( Table 4).

There were statistically significant increases in TW in both maxillary and mandibular arch at the end of treatment with the Damon System ( Table 5) for most measurements, except for the maxillary intercanine distance.

The results of the Pearson correlation coefficient showed statistically significant negative correlations between buccal bone thickness (BBT) and buccal inclination for the right maxillary lateral incisor (r= -0.68; *p*<0.00), left maxillary second premolar (r= -0.64; *p*<0.00), and right mandibular canine (r= -0.64; *p*<0.00) at 4mm from the cementoenamel junction. Statistically significant negative correlations between buccal bone thickness (BBT) and transversal width (TW) were observed for the left maxillary second premolar at 4mm (r= -0.561; *p*= 0.01) and 6mm (r= -0.532; *p*= 0.02) and positive correlation for the mandibular right first premolar at 4mm (r= 0.529; *p*= 0.02).

## Discussion

Among the advantages of the self-ligating system claimed by the manufacturers are low friction, light forces, reduction in the number of extractions, less chair time, and greater appointment intervals (3). However, the literature did not support some of these advantages, including that the light forces applied promote posterior expansion without dental proclination (7-10). Therefore, the present study was designed.

Most teeth showed a decrease in BBT, with greater differences mainly observed for the posterior arch area after treatment ( Table 2, Table 3), corroborating previous findings (7,8). This result suggests that the claims that low forces application induces bone remodeling in the direction of tooth movement were not confirmed. Recently, Alhaija and Taha (15) compared changes in pulpal blood flow between conventional and self-ligating fixed orthodontic brackets during the leveling and alignment stage and showed no significant difference in pulpal blood flow between the brackets tested, suggesting that a similar magnitude of the force was delivered through the slot of the two bracket types.

Regarding buccolingual dental inclinations (BLI), for the maxillary arch, all incisors, right and left first and second premolars showed buccal inclination, and for mandibular arch for most teeth, except the left canine and first molar ( Table 4). Garlock *et al*. (11) also found statistically significant mandibular incisor proclination. This finding does not corroborate the “lip bumper” effect claimed by manufacturers regarding the low pressure from the Damon system and the resting lip pressure decreasing the tendency for incisor proclination.

After treatment, the maxillary right canine and first and second molars were more upright. The molar position findings may be related to the molar lingual inclination tendency in untreated subjects with Class I subjects (16-18). Moreover, our results are in accordance with a previous CBCT study by Billings *et al*. (19) that evaluated changes in BLI and BBT after orthodontic treatment using an edgewise appliance and showed that orthodontic treatment leads to an increase in mandibular molar buccal inclination and a decrease in maxillary molar buccal inclination. Some previous studies (20,21) do not support our findings and observed buccal inclination of the maxillary molars. The differences in these results may probably be related to the measurement protocol and methodology.

Transversal width increase is common in orthodontic treatment performed with self-ligating brackets (12,22-26). Studies have shown a greater transverse increase in patients treated with the Damon System when compared to the conventional bracket system (23,24,26,27). The Damon system arch form, which is more expanded in the premolar segments, may be related to this finding. Using virtual models, Lucchese *et al*. (13) also demonstrated increased arch widths. However, they observed no stability of this alteration after 2 years posttreatment since a tendency to transverse dimension restriction was observed.

The maxillary intercanine distance was the only measure that did not demonstrate a statistically significant increase after treatment ( Table 5). Shook *et al*. (28) also found no significant increase in intercanine distance. Our result is probably related to the fact that the right maxillary canine was more uprighted, and the left canine remained almost with the same inclination after treatment ( Table 4).

In the present study, most CBCT scans were obtained using the i-Cat classic scanner with 0.25 mm voxel size, except for just five posttreatment exams obtained using 3D Accuitomo scanner with 0.20mm voxel size. This factor did not influence either the image visualization or the BBT measurements. According to Menezes *et al*. (6), the measurement of bone plate thickness proved to have similar reproducibility in the different image acquisition protocols. However, the 0.2 mm voxel protocol has produced sharper images than the 0.3 and 0.4 mm voxel protocols.

Among the advantages cited by Damon (3) are that, when using these brackets and broader archwires, it is possible to promote posterior expansion without simultaneous incisor proclination, as low friction occurs and light force is applied. However, the results obtained in the present study do not support this claim since it was observed that arch alignment resulted from transverse expansion, dental tipping, and a decrease in alveolar bone thickness.

## Conclusions

The orthodontic treatment using passive self-ligating brackets demonstrated decreased buccal bone thickness, buccolingual dental inclinations, and increased transversal widths. Therefore, the results obtained in the present study do not support the claim that the passive self-ligating system allows alveolar bone remodeling during arch expansion with tipping control.

## Figures and Tables

**Table 1:** Results of descriptive statistics of the initial and final ages, treatment time and Little irregularity index.

Variable	Mean	S.D.	Minimum	Maximum
Initial age (years) (T1)	14.99	1.27	12.93	17.27
Final age (years) (T2)	17.57	1.15	15.30	20.21
Treatment time (years) (T2-T1)	2.58	0.98	1.07	3.87
MxII (mm)	11.39	5.11	5.35	24.01
Mdll (mm)	8.36	3.56	3.50	17.72

MxII. – Maxillary Little irregularity index MdII. – Mandibular Little irregularity index

**Table 2:** Comparison of buccal bone thickness (BBT) changes at 4 mm level in the maxillary and mandibular arches (dependent t-test) (N=21).

Variable BBT (mm)	Initial (T1)		Final (T2)		BBT change (T2-T1)		p
	Mean	S.D.	Mean	S.D.	Mean	S.D.	
Maxillary arch							
17	1.70	0.81	1.72	0.42	0.02	0.72	0.868
16	1.42	0.51	0.92	0.54	-0.50	0.54	0.001*
15	2.08	0.62	1.50	0.74	-0.57	0.75	0.006*
14	0.96	0.34	0.80	0.57	-0.15	0.49	0.201
13	0.09	0.25	0.00	0.02	-0.08	0.23	0.139
12	0.44	0.51	0.10	0.28	-0.34	0.56	0.021*
11	0.14	0.24	0.13	0.22	-0.01	0.18	0.798
21	0.23	0.35	0.13	0.28	-0.10	0.44	0.365
22	0.32	0.47	0.24	0.37	-0.08	0.51	0.518
23	0.13	0.37	0.03	0.10	-0.10	0.35	0.260
24	0.96	0.52	0.56	0.59	-0.40	0.64	0.021*
25	2.10	0.64	1.54	0.74	-0.55	0.78	0.000*
26	1.31	0.57	1.01	0.60	-0.30	0.54	0.003*
27	1.65	1.10	1.31	0.89	-0.34	0.95	0.161
Mandibular arch							
37	3.62	2.10	3.95	2.60	0.32	3.16	0.679
36	1.12	0.76	0.74	0.47	-0.39	0.60	0.016*
35	0.98	0.38	0.81	0.52	-0.16	0.65	0.317
34	0.35	0.29	0.24	0.23	-0.11	0.27	0.111
33	0.11	0.24	0.09	0.16	-0.02	0.18	0.608
32	0.28	0.50	0.17	0.25	-0.11	0.52	0.372
31	0.32	0.34	0.20	0.23	-0.11	0.32	0.158
41	0.27	0.38	0.22	0.22	-0.04	0.28	0.512
42	0.35	0.70	0.15	0.23	-0.20	0.77	0.289
43	0.10	0.18	0.06	0.17	-0.03	0.16	0.381
44	0.19	0.19	0.30	0.27	0.10	0.27	0.128
45	0.68	0.41	0.76	0.42	0.08	0.54	0.540
46	0.78	0.41	0.65	0.37	-0.13	0.37	0.155
47	4.31	1.95	3.40	2.00	-0.91	1.86	0.006*

*Statistically significant for *p*<0.05.

**Table 3:** Comparison of buccal bone thickness (BBT) changes at 6 mm (maxillary arch) and 8 mm (mandibular arch) levels (dependent t-test) (N=21).

Variable BBT (mm)	Initial (T1)		Final (T2)		BBT change (T2-T1)		p
	Mean	S.D.	Mean	S.D.	Mean	S.D.	
Maxillary arch							
17	2.27	0.91	2.40	0.70	0.13	0.63	0.395
16	1.19	0.56	0.70	0.54	-0.49	0.64	0.006*
15	2.01	0.77	1.62	0.98	-0.38	0.80	0.062
14	0.74	0.95	0.87	0.53	0.13	0.55	0.333
13	0.22	0.27	0.34	0.32	0.12	0.40	0.228
12	1.08	0.83	0.80	0.79	-0.27	0.58	0.070
11	0.57	0.50	0.64	0.58	0.07	0.49	0.534
21	0.86	0.65	0.78	0.62	-0.08	0.59	0.576
22	1.28	0.70	0.90	0.83	-0.38	0.78	0.057
23	0.34	0.44	0.27	0.29	-0.07	0.38	0.420
24	0.81	0.44	1.06	0.59	0.25	0.74	0.180
25	2.10	0.78	1.72	0.97	-0.37	0.80	0.073
26	1.21	0.66	0.72	0.69	-0.48	0.63	0.006*
27	2.18	1.19	2.04	0.72	-0.14	1.05	0.588
Mandibular arch							
37	4.10	3.85	5.22	2.92	1.11	3.86	0.250
36	1.97	1.44	1.40	0.98	-0.57	1.26	0.080
35	1.75	1.35	1.21	0.84	-0.54	1.05	0.048*
34	0.73	0.78	0.76	0.58	0.02	0.69	0.863
33	0.40	0.48	0.31	0.31	-0.07	0.39	0.442
32	0.61	0.54	0.78	0.68	0.17	0.69	0.328
31	0.90	0.76	1.03	1.06	0.12	0.90	0.562
41	0.69	0.62	0.88	0.92	0.18	0.85	0.375
42	0.55	0.60	0.63	0.75	0.08	0.59	0.574
43	0.24	0.29	0.14	0.18	-0.10	0.34	0.246
44	0.67	0.57	0.58	0.45	-0.08	0.66	0.591
45	1.34	0.90	1.24	0.58	-0.10	0.78	0.583
46	1.97	0.91	1.24	0.74	-0.72	0.79	0.001*
47	4.25	3.97	5.24	2.70	0.99	3.95	0.315

*Statistically significant for *p*<0.05.

**Table 4:** Comparison of buccolingual dental inclinations (BLI) changes in the maxillary and mandibular arches (dependent t-test) (N=21).

Variable BLI (^0^)	Initial (T1)		Final (T2)		BLI change (T2-T1)		p
	Mean	S.D.	Mean	S.D.	Mean	S.D.	
Maxillary arch							
17	82.69	5.47	79.96	6.73	-2.72	5.43	0.055
16	79.01	5.75	77.14	5.09	-1.87	5.19	0.157
15	80.41	7.58	84.77	6.26	4.35	6.52	0.014*
14	80.85	8.05	87.81	3.98	6.95	6.80	0.000*
13	97.20	8.79	94.40	5.45	-2.80	8.37	0.186
12	100.25	8.84	106.60	4.61	6.35	7.81	0.004*
11	107.03	5.52	110.04	3.74	3.00	4.00	0.006*
21	106.78	6.14	111.51	3.74	4.73	5.37	0.002*
22	100.16	10.54	109.02	3.80	8.86	11.91	0.007*
23	96.70	10.56	96.96	3.92	0.26	9.82	0.912
24	81.55	7.12	89.65	5.97	8.10	7.64	0.000*
25	83.51	9.47	88.87	5.22	5.35	6.52	0.003*
26	82.46	7.57	79.95	7.47	-2.50	6.99	0.158
27	86.25	8.94	84.02	8.27	-2.22	5.52	0.115
Mandibular arch							
37	59.52	7.82	63.77	8.42	4.24	6.64	0.018*
36	62.70	5.74	64.21	4.91	1.51	6.22	0.331
35	68.10	5.93	71.00	5.35	2.90	3.55	0.003*
34	69.74	8.81	78.14	4.36	8.34	7.71	0.000*
33	85.53	9.58	85.68	4.56	0.15	7.20	0.931
32	89.17	8.29	97.62	5.13	8.44	6.52	0.000*
31	94.73	8.28	100.01	5.12	5.28	6.14	0.002*
41	93.49	7.30	100.32	4.45	6.83	5.59	0.000*
42	89.31	7.77	98.14	3.87	8.83	7.64	0.000*
43	85.22	7.27	89.56	4.61	4.33	6.98	0.020*
44	71.60	7.43	78.88	3.35	7.27	6.75	0.000*
45	67.10	6.98	72.57	4.09	5.47	5.51	0.000*
46	60.61	6.04	63.49	5.29	2.87	3.74	0.005*
47	56.19	5.88	61.92	7.16	5.73	7.62	0.006*

*Statistically significant for *p*<0.05.

**Table 5:** Comparison of transversal widths (TW) changes in the maxillary and mandibular arches (dependent t-test) (N=21).

Variable TW (mm)	Initial (T1)		Final (T2)		TW change (T2-T1)		p
	Mean	S.D.	Mean	S.D.	Mean	S.D.	
Maxillary arch							
7-7	57.92	3.17	59.82	2.97	1.90	1.42	0.000*
6-6	52.98	3.44	54.88	2.65	1.90	1.54	0.001*
5-5	46.48	4.35	50.69	2.63	4.21	2.51	0.000*
4-4	41.42	2.05	45.82	2.04	4.40	1.55	0.000*
3-3	37.29	6.62	38.74	1.54	1.44	6.23	0.352
Mandibular arch							
7-7	51.95	4.24	54.34	3.89	2.39	2.82	0.002*
6-6	46.57	3.56	48.71	3.41	2.13	2.19	0.000*
5-5	40.22	4.20	42.98	2.33	2.75	3.72	0.007*
4-4	34.12	3.09	38.09	2.42	3.96	2.10	0.000*
3-3	27.77	3.81	29.94	1.31	2.17	3.74	0.029*

*Statistically significant for *p*<0.05.

## Data Availability

The datasets used and/or analyzed during the current study are available from the corresponding author.
